# Evaluation of posture in four different robotic surgical systems

**DOI:** 10.1007/s00464-025-12136-y

**Published:** 2025-09-08

**Authors:** Dolores T. Krauss, Stefanie Brunner, Jennifer A. Eckhoff, Christian Storms, Benjamin Babic, Thomas Schulz, Lorenzo Macone, Jun Kinoshita, Kengo Hayashi, Saki Hayashi, Thomas Schmidt, Christiane J. Bruns, Hans F. Fuchs

**Affiliations:** 1 Department for General, Visceral, Thoracic and Transplant Surgery, Faculty of Medicine and University Hospital Cologne, University of Cologne, Kerpener Str. 62, 50937 Cologne, Germany; 2 Department for General and Visceral Surgery, Sana Klinikum Offenbach, Offenbach, Germany; 3 Department of General and Visceral Surgery, St. Elisabethen Krankenhaus, Frankfurt, Germany; 4 Department of Gastroenterological Surgery, Ishikawa Prefectural Central Hospital, Kuratsuki Higashi 2-1, Kanazawa City, Ishikawa, 920-8211 Japan

**Keywords:** Robotic surgery, Ergonomics, DaVinci surgical system, Dexter surgical system, Hinotori surgical system, Hugo surgical system

## Abstract

**Background:**

While many ergonomic challenges traditionally faced in open and laparoscopic surgery have been overcome by robotic surgery, new challenges have been created. This study aims to identify and compare the ergonomic characteristics of a variety of robotic systems to ultimately lay the foundation for ergonomic guidelines.

**Methods:**

Measurements evaluating the surgeon and their interaction with the new technology were applied in either a laboratory or a real-life setting. A video camera was used to capture the surgeon at the console during surgery. For evaluation of the images, axes were placed along the joints of the surgeon. Corresponding angles were calculated based on the position of the individual axes in relation to each other and compared to recommendations for a healthy workspace.

**Results:**

A total of 13 surgeries were measured during actual cases using the DaVinci with a total of *n* = 20,250 pictures used for analysis; 2 surgeries were measured using the Dexter, with a total of 1,994 pictures used for analysis; 17 measurements were taken with the Hugo™ RAS System in a simulation setting, with a total of 1,179 pictures used for analysis. In addition, a total of 327 pictures from a simulation setting were used for analysis of the Hinotori. Corresponding angles for knee, elbow, and back were within recommendations for all systems; hip angles were not within recommendations for any system, and the neck posture was only within recommendations for the Hinotori.

**Conclusion:**

A variety of different robotic systems differ in ergonomic requirements. The neck can be identified as an area of need for improvement for most systems. Future studies, particularly in a randomized controlled fashion, need to be performed to further analyze ergonomic features of the different systems, and guidelines displaying the variety of systems need to be implemented to create a healthy and safe workplace.

Ergonomics play a crucial role in maintaining endurance, efficiency, and a long and healthy work life in a surgical environment. Surgical ergonomics, however, vary and lack universal guidelines, despite rapid technological advancements and the introduction of new robotic techniques. A study by Meltzer et al. utilized wearable technology to measure the ergonomic risks faced by surgeons performing open surgery, creating a risk scale based on the percentage of time spent in each risk category [1]. Applying this risk scale to the robotic surgeon console reveals high-risk postures and a need for improvement in the robotic work environment. A comprehensive ergonomic study conducted by Lee et al. demonstrated a reduction of physical demands during surgery when a robotic device is used; however, it also found that 56% of robotic surgeons regularly experienced work-related discomfort [2]. In a systematic review by Epstein et al., 60% of surgeons reported a 12-month prevalence for the development of neck pain, 52% reported shoulder pain, with the overall career prevalence for degenerative lumbar spine disease being 19% and for rotator cuff pathology being 18%, further highlighting the relevance of this topic [3]. While robotic surgery has decreased back and shoulder pain, still 28% of surgeons complained of neck and finger pain, showcasing that still every fourth surgeon leaves the workplace with physical discomfort [4].

While the plethora of robotic cases over the past two decades has been performed using DaVinci systems, there have been a number of new robotic systems from different manufacturers which have entered the market or are currently seeking FDA approval. Each of these new systems promotes different surgical advantages in addition to seeking to improve surgeon comfort through enhanced ergonomic features. However, studies and hence evidence on these differences as well as ergonomic benefits and challenges require objective measurements and are currently lacking. This study seeks to identify the ergonomic characteristics of various robotic systems to help develop guidelines and improvements for a healthier workspace in the robotic operating room.

## Material and methods

**Fig. 1:**
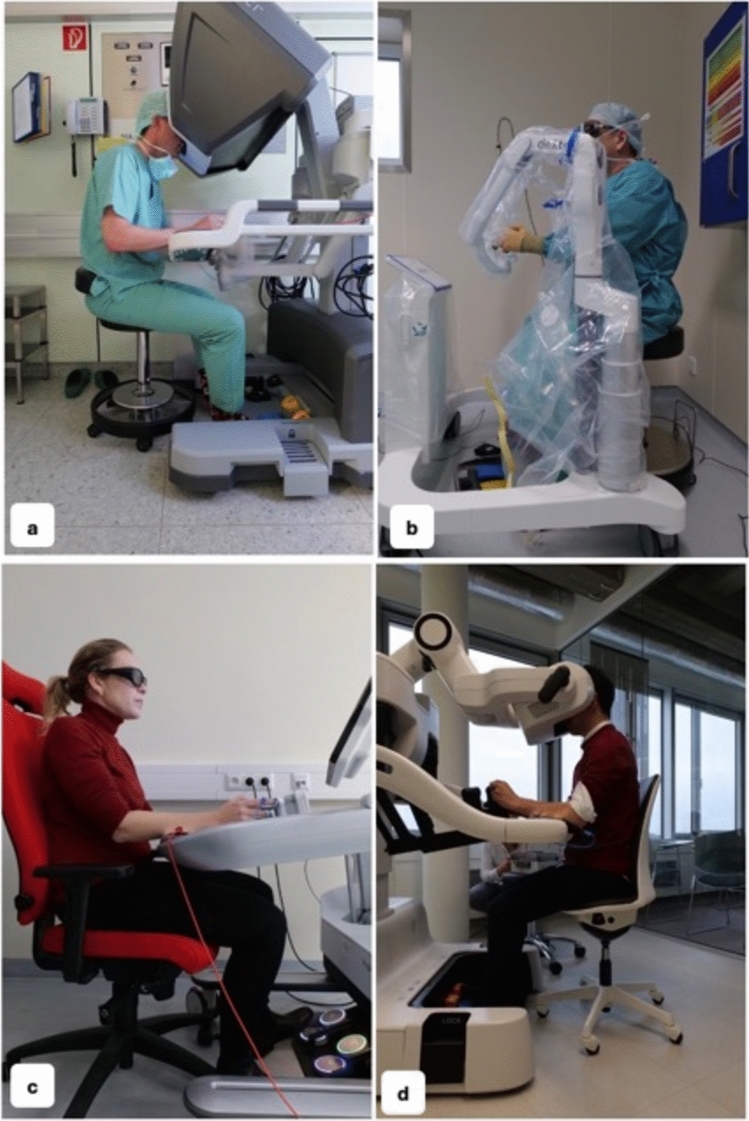
Measurements evaluating the surgeon and their interaction with the new technology were performed in four different robotic systems – **a** DaVinci, **b** Dexter, **c** Hugo, **d** Hinotori

A prospective, non-blinded, non-randomized, open clinical monocenter study to identify the ergonomic challenges in different robotic systems was performed (IRB 21-1274_1). Measurements evaluating the surgeon and their interaction with the new technology were applied in either a laboratory or an operating theater during actual surgical cases (Fig. 1).

### Study participants

Board certified surgeons that were experienced in robotic-assisted minimally invasive surgery were included in the study. Additionally, only surgeons who were sufficiently trained and experienced in the use of the respective system were used for this evaluation. This included having sufficiently completed the manufacturers’ training pathway. A total of 3 different surgeons were measured (1 for the da Vinci system, 1 for the Dexter system, 1 for the Hinotori system). The expert for the Hugo system was also part of the da Vinci expert group. Informed consent was obtained, written and verbally.

### Da Vinci Xi

Measurements of the da Vinci Xi system (Intuitive Surgical, Sunnyvale, CA, USA) were performed during the ERASE Trial (Evaluation of Ergonomics and Surgeon’s Stress Levels during Robotic Assisted Minimally Invasive Esophagectomy (RAMIE): The ERASE Trial – submitted for publication) in a real-life setting.

The da Vinci system is generally considered to be the most widely recognized and clinically available robotic surgical system on the market. It comprises a patient cart, a surgeon console, and a video cart. The video cart includes a generator for electrosurgical instruments and a screen for the bedside assistant and staff in the operating room (OR). Ergonomic challenges may be faced as the bedside assistant’s instruments collide with robotic arms or limitations in movement may create ergonomic issues for the assistant throughout the procedure. Additionally, due to the fixed location of the video cart, which is determined by the length of the cables and limited space, there can often be a discrepancy between the working and visual axis for the bedside assistant [5].

Conversely, the surgeon console can be adjusted in various ways to support an ergonomically favorable posture, and settings can be saved to a username for easy retrieval. As working at the da Vinci surgeon console is similar to working at a microscope, recommendations for an ergonomic posture are easily applicable. The space above the binoculars is padded, allowing the forehead to rest comfortably, although this creates a neck posture that does not align with current ergonomic recommendations. The console features a 3D high-definition (HD) display that does not require additional 3D glasses and eliminates human tremor, thus improving visualization. Additionally, the console includes an arm rest and foot pedals for camera guidance and the application of electrosurgical instruments.

### Dexter robotic system

Evaluation of the Dexter (Distalmotion, Epalinges, Switzerland) was performed in a real-life setting while performing robotic-assisted minimally invasive cholecystectomy. The Dexter Robotic System, developed in Switzerland and introduced in 2021, is CE certified and FDA approved. It is a modular platform and integrates robotic and laparoscopic techniques, allowing for interchangeable use during procedures, thereby optimizing workspace utilization. The Dexter system comprises a sterile surgeon console, two patient carts equipped with robotic arms, and a robotic endoscope arm compatible with all endoscopic systems. The compatibility of laparoscopic instruments and this robotic system allows a transition between both modalities without the need for redocking or recalibration [6].

The console can be used in a sterile or non-sterile fashion, allowing the surgeon to switch between robotic and laparoscopic techniques within 15 to 30 s while maintaining sterility throughout the procedure [7]. The open surgeon console as well as the sterile usage may enhance communication of the lead surgeon with the team as well as the bedside assistant; however, surgeons have reported an increase of distractions using an open console design. Furthermore, the console may be adjusted in height; however, it is not yet equipped to save the setting for future application. As the energy application is not built into the system but used as a separate component, the foot pedal needs to be adjusted manually, making it time-consuming to follow ergonomic needs [8].

### Hugo

The Hugo™ RAS System (Medtronic, Dublin, Ireland) was evaluated in a simulation setting during the CEMRobSurg Study (Cologne ergonomic measurement for robotic surgery using the Hugo™ RAS System)[9].

Similar to other, newer robotic systems, the Hugo™ RAS System employs a modular design. The system can function as a complete robotic surgical system with 3–4 instrument modules or as an assistant for laparoscopic surgery with a single instrument module. The console features an open design and is equipped with a 3D HD display that necessitates the use of 3D glasses. While the console can be adjusted to accommodate the surgeon’s ergonomic needs, it as well lacks the capability to save these settings for future procedures. Given the fast-paced and demanding nature of a surgeon’s daily routine, the adjustment of the console to establish an optimal ergonomic workspace may occasionally be overlooked. Additionally, it is outfitted with six foot pedals for camera guidance and the operation of bipolar and monopolar instruments, presenting a cognitive and ergonomic challenge to accurately identify and activate the appropriate pedal for each specific application.

### Hinotori

Measurements of the hinotori™ robotic surgical system (Medicaroid Corporation, Kobe, Japan) were performed in a simulation setting analogous to the measurements with the Hugo system.

The Hinotori features an arm with eight articulated joints, providing an additional degree of freedom compared to the DaVinci system [10–12]. The system also adopts a docking-free design, which eliminates the need for docking between robotic arms and trocars [10–13]. This innovation minimizes excessive traction on the abdominal wall, thereby reducing the risk of tissue damage [14].

The surgeon’s cockpit is designed similarly to the one from the da Vinci Xi system, including binoculars providing the surgeon with a 3D view without the need for specific glasses. An additional adjustment of the headpiece may be made, creating an optimal posture for the neck region while making it easy for the surgeon to obtain a good view through the lenses [11].

Table 1 summarizes the ergonomic features of the surgeon’s console of the respective systems.

**Table 1:** Ergonomic features, challenges and configurations of the surgeon’s console of different robotic systems

	da Vinci Xi	Dexter	Hugo™ RAS system	hinotori™
Components of the system	Patient cart, surgeon console, video cart	2 robotic modules, 1 camera module, surgeon console	3 robotic modules, 1 camera module, surgeon console, video cart	Patient cart, surgeon console, video cart
Console design	Closed	Open + use of external video tower	Open with own screen	Closed
Food pedals	X	(X)	X	X
Arm rest	X	X	X	X
Visualization	3D without glasses	3D or 2D (individual video tower)	3D with glasses	3D without glasses
Ability to save console settings	X	X	X	X
Ergonomic features	Adjustability and easy retrieval for future surgeries	Open design, sterile use for easy switch between techniques	Open design with integrated screen	Adjustability of the head piece to facilitate a low-risk neck posture
Ergonomic challenge	High risk neck posture due to padded area for forehead	Settings not saved, foot pedals need to be manually adjusted	Settings not saved, mandatory 3D glasses, complexity of foot pedals	Closed design may hinder communication

### Posture analysis

**Fig. 2:**
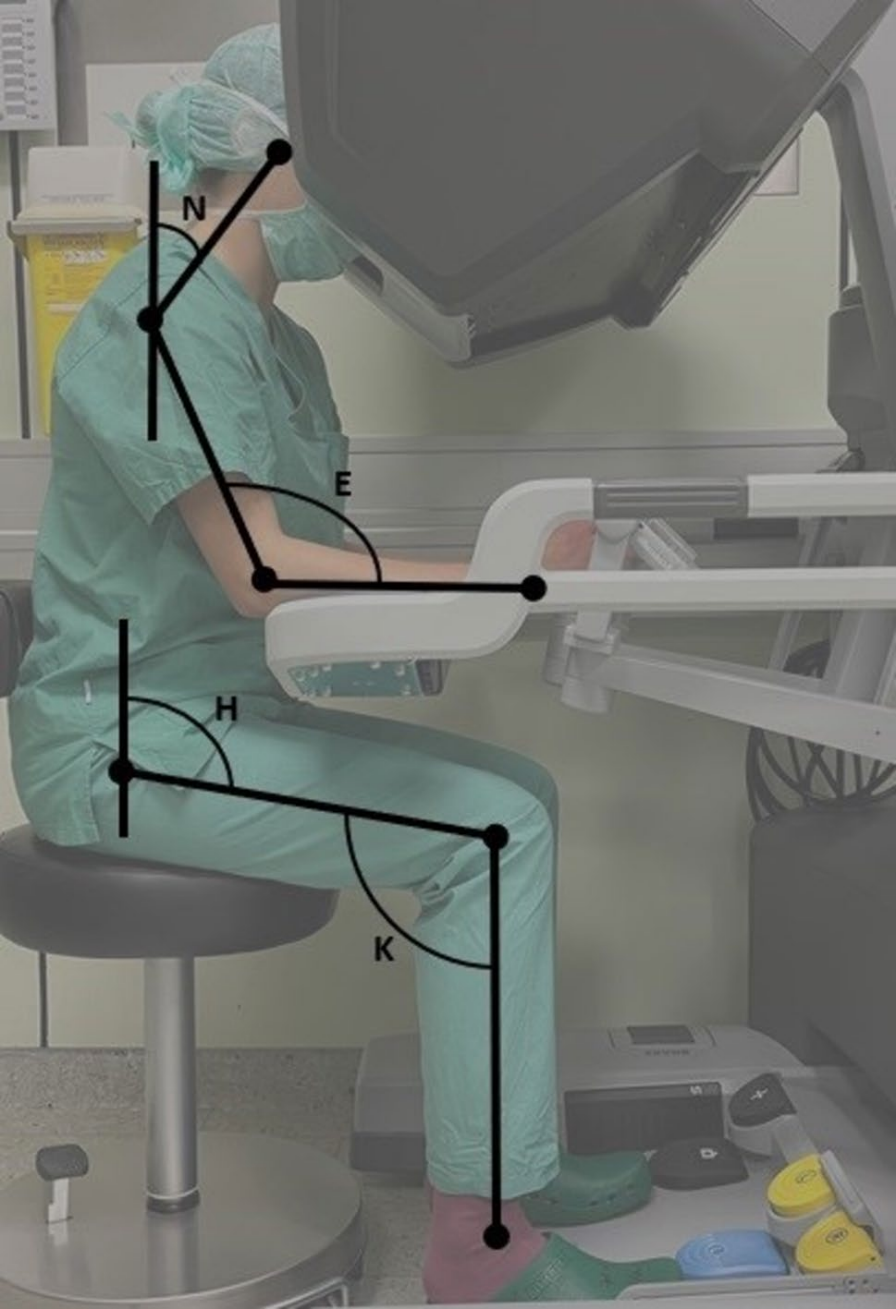
Corresponding angles for the evaluation of posture are calculated based on the axes’ positions

A video camera captured the surgeon at the console during surgery or simulation. Positioned at a 90° angle, the camera ensures the console armrest appears horizontal. Axes are placed through the surgeon’s joints (torso, neck, shoulder, hip, and knee) for image evaluation. Corresponding angles are calculated based on the axes’ positions and compared to healthy workspace recommendations (Fig. 2). The authors have published a detailed report on the methods of this analysis previously [9]. For optimal console positioning, the surgeon should bend their knees at 90°, with feet resting on the floor or on the pedals as needed. Upper arms should be perpendicular to the floor, forearms resting neutrally on the armrests, and elbows tucked to the body at a 90° angle. The ideal position allows easy pedal access, comfortable forearm rest, and a clear view of the screen without excessive neck (10–15°) or back bending (0–10°). A hip angle of 110–120° is recommended [1, 15]. Statistical comparison and analysis were performed using SPSS Statistics (IBM, New York, USA). Mean, median values, and ranges are reported.

## Results

A total of 13 surgeries were measured during a robotic-assisted esophagectomy using the da Vinci with a total of *n* = 20,250 pictures used for analysis. A total of 2 cholecystectomies were measured using the Dexter robot with a total of 1,994 pictures used for analysis. A total of 17 measurements were taken with the Hugo™ RAS System in a simulation setting with a total of 1,179 pictures used for analysis. A total of 327 pictures from a simulation setting were used for analysis of the Hinotori.

Results from the evaluation of posture are depicted in Tables 2 and 3. It shows a comparison of the results to the current recommendations for an ergonomic posture. Corresponding angles for knee, elbow, and back were within recommendations for all systems. The corresponding hip angles were not within recommendations for any system. The neck posture was only within recommendations for the Hinotori.

**Table 2:** Mean, median, and range of posture for the variety of systems

DaVinci							
	Knee	Hip	Shoulder	Neck	Elbow	Back	Forearm
Mean (°)	119.4	102.3	25.1	40.7	102.2	7.5	6.7
Median (°)	120.9	101.7	24.2	40.8	101.1	7.4	5.9
Range (°)	141.8 – 73	114.9 – 70.8	49.3 – 6.6	57.3 – 13.2	135.7 – 79.7	17.6 – 0	34.3—0
Dexter							
	Knee	Hip	Shoulder	Neck	Elbow	Back	Forearm
Mean (°)	117.8	102.3	45.3	33.3	119.6	1.5	14.1
Median (°)*	N/A	N/A	N/A	N/A	N/A	N/A	N/A
Range (°)	110.8 – 124.8	98.1 – 106.5	43.5 – 47	32.2 – 34.4	113.1 – 126.1	1.1 – 2	8.9 – 19.3
Hugo							
	Knee	Hip	Shoulder	Neck	Elbow	Back	Forearm
Mean (°)	101.1	102.5	25.3	30.5	113.8	9.3	7.9
Median (°)	92.7	98.2	27.9	31.4	117.2	9	7
Range (°)	74.2 – 142	81.8 – 124–2	5–1 – 40.4	17.1 – 47.1	85.6 – 133.9	0.4 – 20.6	0.6 – 16.8
Hinotori							
	Knee	Hip	Shoulder	Neck	Elbow	Back	Forearm
Mean (°)	111.1	95	30.8	11.3	99.2	5.7	15.9
Median (°)*	N/A	N/A	N/A	N/A	N/A	N/A	N/A
Range (°)*	N/A	N/A	N/A	N/A	N/A	N/A	N/A

^*^Due to limited data, no median/range was calculated

**Table 3:** Comparison of the results to the current recommendations for an ergonomic posture

	Knee	Hip	Shoulder*	Neck	Elbow	Back	Forearm
Recommendation (°)	≥ 90	110–120	N/A	10–15	≥ 90	0–10	N/A
DaVinci							
Within recommendation	Yes	No	N/A	No	Yes	Yes	N/A
Dexter							
Within recommendation	Yes	No	N/A	No	Yes	Yes	N/A
Hugo							
Within recommendation	Yes	No	N/A	No	Yes	Yes	N/A
Hinotori							
Within recommendation	Yes	No	N/A	Yes	Yes	Yes	N/A

^*^ No recommendation for a corresponding shoulder angle was available for comparison

## Discussion

Our study identified an ergonomic low-risk posture for the knee, elbow, and back for all four robotic systems, supporting the statement that robotic surgery provides improved ergonomics for the surgeon. However, this study was also able to show the need for ergonomic improvement for the hip and neck region, with the neck region being a differentiating factor between robotic systems.

In line with our findings, a pilot study from Lawson et al. comparing laparoscopic and robotic bariatric surgery indicated that robotic surgery caused more neck discomfort, whereas laparoscopic surgery increased shoulder and upper back discomfort. The RULA (rapid upper limb assessment) score revealed less ergonomic arm and wrist positions in laparoscopic surgery, while trunk posture was less ergonomic in robotic surgery [16]. A study by Armijo et al. used EMG to assess objective muscle effort alongside a validated fatigue scale for subjective assessment during robotic-assisted and laparoscopic surgery. The results showed no differences in self-reported fatigue between the two surgical approaches. However, consistent with our findings, the robotic approach was associated with increased muscle activation of the trapezius muscle, indicating strain on the neck [17]. A study conducted by Yu et al. utilized wearable motion tracking sensors to objectively assess the posture of surgeons during robotic-assisted surgery. The console surgeon was observed to have more static postures and elevated unsupported shoulder postures, indicating an insufficient use of the armrest [18].

While those findings and our study focused on providing quantitative data to support the notion that robotic surgery provides improved ergonomics, other studies concentrated on subjective data. A survey by Plerhoples et al. revealed a significant reduction in physical discomfort, from 55.4 to 8.3%, associated with robotic surgical approaches compared to laparoscopic or open techniques. Additionally, an increased caseload in the laparoscopic group correlated with heightened physical discomfort, whereas no such correlation was observed in the robotic group [19]. A study by Lee et al. with 432 robotic surgeons, however, found that despite ergonomic improvements, 56.1% experienced physical discomfort during robotic surgery. Interestingly, surgeons confident in their ergonomic settings reported less discomfort [2]. However, as these data are subjective, as well as the surgeon’s evaluation of comfort, statements from those studies are limited.

While these data objectively and subjectively support the need for further research, it is important to mention that current evidence on ergonomics in robotic surgery solely focuses on the most commonly used da Vinci system. Research on other robotic systems is limited due to their recent introduction; however, it may reveal ergonomic benefits in future.

The Hinotori system, for example, has a surgeon cockpit designed with ergonomic principles such as a flexible three-dimensional viewer that is expected to reduce the physical burden on the surgeon more effectively than other platforms [20]. Additionally, the docking-free system provides ample space around the trocars, enabling assistants to work more easily and reduce stress [10]. Furthermore, the collision alarm function helps prevent the arm from colliding with the assistant, other robotic arms, or the patient, which has the potential to alleviate the assistant’s stress and promote better posture [11]. However, this feature also presents issues related to operability and excessive operation stoppage caused by the safety sensors, which require further development [20]. While initial findings suggest notable ergonomic advantages, robust evidence is still lacking. Medicaroid emphasizes the system’s design to reduce surgeon fatigue and improve precision; however, further studies are needed to confirm its ergonomic superiority in robotic surgery.

The Dexter in comparison provides an ergonomic design of the robotic arms, allowing them to be folded and maintaining a laparoscopic home state and enabling quick access to the patient. This space-saving feature is a notable improvement over traditional robotic systems, which often obstruct access to the surgical field when conversion is necessary. Additionally, the Dexter system permits customizable trocar setups similar to classic laparoscopic arrangements, enhancing maneuverability and access during surgery. This flexibility supports optimal positioning for the main surgeon, assistant, and scrub nurse [21]. The open console and modular design of the Dexter Robotic System may provide significant advantages for surgeons, facilitating a tailored approach to various procedural demands while enhancing accessibility and efficiency in the operating room.

A previous study from our group focused on the ergonomics of the newly introduced Hugo robotic system. Measuring the interaction of subjects of different levels of experience, we were able to provide a detailed report on the ergonomics of this new system. Interestingly, while medical students without previous robotic experience showed high-risk postures for the neck and elbow, high-risk postures of the robotic experts were mostly found for the knee and hip region [9]. While ergonomics in the OR do not only affect the lead surgeon, a study from Olsen et al. focused on the impact of the introduction of a new system on the team and especially on the scrub nurses. Contrary to what is commonly claimed, that new robotic systems are easy to understand and the setup is quick to learn, this study revealed a total of 20 cases needed until the setup time significantly decreased [22]. While 20 cases do not seem like a lot of cases, due to staff rotation, a long time is needed to thoroughly train a whole team. Another study from Mintz et al. reported the first series of patients undergoing a heller myotomy using the new Hugo robotic system. In addition to the procedure being safely performed, the authors report an improvement in docking angles, maneuverability, and surgeon ergonomics at the console [23]. The open design of the Hugo and also the Dexter system is supposed to improve surgeon ergonomics, as well as facilitate communication between the lead surgeon and the team in the OR. However, while descriptions of the systems and first experiences of surgeons mention those improvements, actual quantitative evidence is not readily available in the literature.

**Fig. 3:**
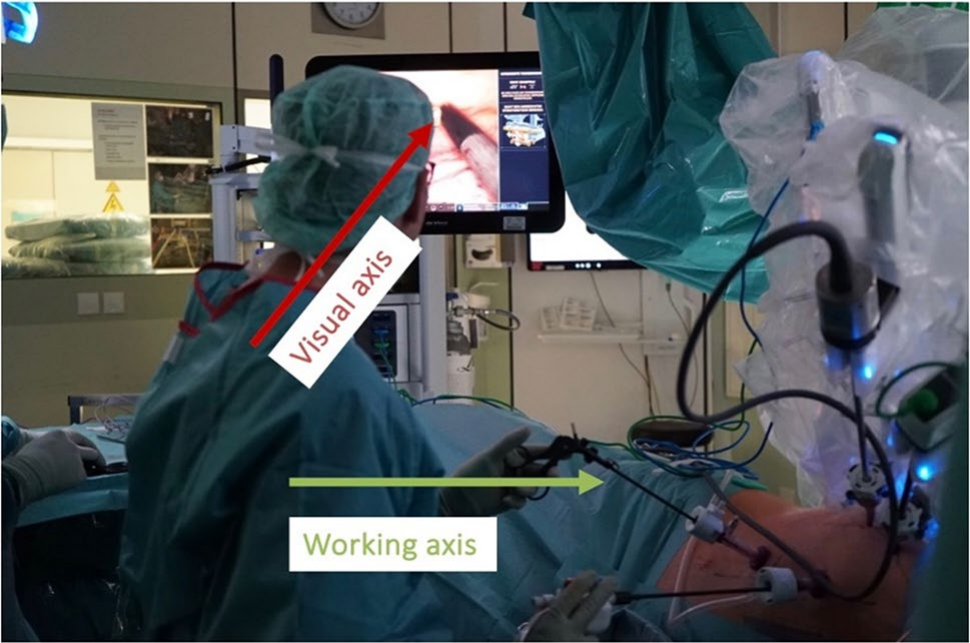
A discrepancy between the working axis and the visual axis can lead to ergonomic challenges, especially for the bedside assistant

Another fact we did not focus on in this study is bedside assistant’s ergonomics. In brief, the major difference between the perfect visualization of the console surgeon with an aligned working and visual axis and the impaired visualization of the bedside assistance with a concerning discrepancy of the working and visual axis further showcases the importance of this topic. Further studies from our department are currently focusing on benefits and problems of the bedside assistant surgeon. Figure 3 shows the dilemma.

As an ergonomic setup of the OR, especially when performing a minimally invasive surgery, has been identified as a major challenge, training in ergonomics has become more important than ever. Furthermore, raising early awareness of a healthy workspace in trainees is crucial, as they may experience even more physical strain than experienced surgeons, and correcting high-risk postures early may lead to a long and healthy work life [24]. A survey conducted by Franasiak et al. revealed that only 16.6% of surgeons had previously participated in ergonomic training; all found such to be very helpful, and even 88% changed their practice accordingly [25]. In line with this, a study from Van’t Hullenaar evaluated the effect of ergonomic training while using a robotic simulator, showing a significant decrease in unnecessary hand movement, smaller deviation from a neutral position, and, most importantly, a significant improvement in ergonomics, while no change for subjective scores or performance was observed [26]. Showing the impact of ergonomic training within residents in robotic surgery, Castaldi et al. demonstrated a reduction of 51% in console time after successful application of an educational video in addition to peer hands-on training [27].

A structured and comprehensive ergonomic training curriculum by industry or within robotic training courses, however, is yet lacking. Industry-sponsored trainings and first introduction to the systems include a systematic introduction to the surgeon’s console as well as its functions. During hands-on training, the adjustments of the console are emphasized and roughly corrected if an ergonomically high-risk posture is seen. However, a further ergonomics training, especially by an ergonomist, is lacking.

Our study is limited by the number of participating surgeons, as only a few selected surgeons were included as experts in their field and for the respective devices. As the new robotic devices become more commonly used, future studies can focus not only on including more surgeons and especially ones from different levels of training, but also on measurements in complex and simple procedures with different durations. Furthermore, this study provides an interindividual analysis limiting comparability of data. However, as hospitals usually do not offer more than one type of robotic device and learning curves as well as trainings are time-consuming, intraindividual analyses are challenging and may even never be possible. As devices obtain CE and FDA approval, studies in a randomized controlled fashion including multiple robotic devices using the same study setup, patients, and surgeons may be performed to overcome this limitation. In addition, the impact of the OR environment may play a key role in the evaluation of ergonomics, as, for example, the OR setup differs between the usage of robotic devices as well as the chair used by the console surgeon. Future studies need to take this into account. To our knowledge, this is the first study that offers quantitative ergonomic insight for the Hinotori and Dexter systems as well as the first comparison of different robotic devices.

## Conclusion

It is undisputable that new robotic systems have improved ergonomics in minimally invasive surgery significantly. A variety of different robotic systems offer not only a variety of technical features and advantages but also differ in ergonomic requirements. As there is still an urgent need to improve ergonomics even in the robotic OR, with especially the neck being identified as an area of concern in this study, it should be recommended to the surgeon to familiarize themselves with the ergonomics of the respective console and device before usage, making sure the setup displays an ergonomically low-risk usage of the system. This may only take a few minutes each day but may convert the robotic OR to a healthier workspace on an everyday basis. Future studies, especially in a randomized controlled fashion, need to be performed to further analyze ergonomic features of the different systems, and guidelines displaying the variety of systems need to be implemented to create a healthy and safe workplace.
